# Topography and clinical features of iris melanoma

**DOI:** 10.1186/s12886-021-02236-3

**Published:** 2022-01-03

**Authors:** Jørgen Krohn, Kristoffer Våge Sundal, Torbjørn Frøystein

**Affiliations:** 1 Department of Clinical Medicine, Section of Ophthalmology, University of Bergen, Bergen, Norway; 2 Department of Ophthalmology, Haukeland University Hospital, N-5021 Bergen, Norway; 3 Department of Oncology and Medical Physics, Haukeland University Hospital, Bergen, Norway

**Keywords:** Distribution, Imaging, Iris melanoma, Location, Topography, Uveal melanoma

## Abstract

**Background:**

To characterise the topographical and clinical features of primary iris melanoma and to visualise the patterns of tumour extent in the iris.

**Methods:**

Clinical characteristics of iris melanomas were analysed, and data on their size, shape, and location were converted into a database of two-dimensional iris charts by means of computer-drawing software. The geometric centre of each tumour was entered into corresponding sectors of the chart. The extent of the melanomas was computationally visualised by merging the iris drawings and displaying the number of overlapping tumours on colour-coded iris maps.

**Results:**

Twenty-nine patients (18 females and 11 males) with a mean age of 52 years met the inclusion criteria. The mean largest tumour diameter was 6.1 mm (range, 1.8–11.0 mm). Five tumours (17%) involved the pupillary margin, 10 (34%) involved the iris root, and 10 (34%) involved both sites. The hemispheric location of the tumour centroid was superior in 3 eyes (11%) and inferior in 25 (89%) (*p* < 0.0001), and the distribution between the temporal and nasal hemispheres was 17 (61%) and 11 (39%), respectively (*p* = 0.26). In females, the iris melanomas were located more temporally (*p*  =  0.02) and had more often originated from a pre-existing naevus (*p* = 0.03), than in males. There was also shift towards more temporally located melanomas in younger patients.

**Conclusions:**

The lower temporal iris quadrant is the preferential area of melanoma occurrence and growth. Iris melanoma tends to be more temporally located in females, who compared with males also have a higher proportion of melanomas arising from a pre-existing naevus.

## Background

The most common primary intraocular malignancy in adults is uveal melanoma, which can arise in any part of the uveal tract from the iris to the ciliary body and choroid. The choroid and ciliary body are the most common sites for uveal melanoma, accounting for 95-98% of the cases, whereas only 2-5% are located to the iris [1–3]. Iris melanomas occur more frequently in Caucasians with light coloured irides and skin compared with other ethnicities. Age-adjusted incidence rates of iris melanoma in Norway have been reported to be 0.10 for females and 0.09 for males per 100,000 person-years, without any significant gender predilection [4]. Patients with iris melanoma tend to have a younger age at diagnosis (typically between 45 and 65 years) and a lower risk of metastasis (about 5% at 10 years) compared with patients with posterior uveal melanoma [3, 5, 6].

The majority of iris melanomas develop from a pre-existing naevus and presents as a variably pigmented, elevated, solitary mass in the iris stroma [7]. Other rarer forms of presentation are diffuse iris melanoma, ring melanoma and tapioca melanoma, the latter being characterised by a pale nodular surface resembling tapioca pudding [8]. Although many previous studies have shown that iris melanoma has a predilection for the inferior part of the iris [7, 9], detailed data on their topographical distribution are scarce. The main objectives of the present study were to characterise the clinical and topographical features of iris melanoma and to visualise the patterns of tumour extent by software assisted tumour mapping.

## Materials and methods

### Study design and patients

This study was conducted at the Department of Ophthalmology at Haukeland University Hospital, which is a tertiary referral centre for ocular oncology in Norway. A search in the institutional database was performed to find patients diagnosed with iris melanoma between January 1988 and June 2019. The patients were identified utilising the International Classification of Diseases diagnosis codes for all diagnoses of iris and ciliary body tumours and procedure codes for iris surgery and related procedures. A total of 462 patients met the search criteria, and their medical records were then screened for the correct tumour diagnosis. Only patients with primary iris melanoma confirmed by biopsy and/or documented growth and records containing detailed descriptions of the size and location of the tumour, as well as slit-lamp photographs and/or iris drawings, were included in the study. The clinical information extracted from the records included gender, age at diagnosis, laterality, referral reasons, symptoms, intraocular pressure (IOP), iris colour, type of treatment, and length of follow-up.

The study was registered and approved by The Regional Committee for Medical and Health Research Ethics, Western Norway (ref. no. 2019/34), and conducted in accordance with the Declaration of Helsinki. All included living patients provided written informed consent for medical record review.

### Tumour data

Data regarding morphological and topographical characteristics of the iris melanomas were obtained from the medical records and careful examination of all available slit-lamp photographs and iris drawings. The data collected included largest tumour diameter and height measured by slit-lamp examination and/or ultrasound biomicroscopy (UBM), tumour margins (pupil, mid-zone, iris root/angle), tumour configuration (flat, dome, nodular, diffuse, ring pattern), tumour pigmentation (amelanotic or pigmented, i.e., subjectively graded as weak, moderate, marked, or partial), ectropion uveae, pupillary distortion, tumour extension (ciliary body, extrascleral), biopsy type, and histopathology.

### Tumour location

The location of the iris melanomas was determined according to their geometric centre (tumour centroid), which for the round and oval lesions corresponded to the midpoint of the largest tumour diameter. For irregular tumours, the location was estimated based on their centremost point. The melanomas were then categorised according to their location in quadrants and hemispheres, defined by a horizontal and vertical line passing through the centre of the pupil.

### Iris mapping

**Fig. 1:**
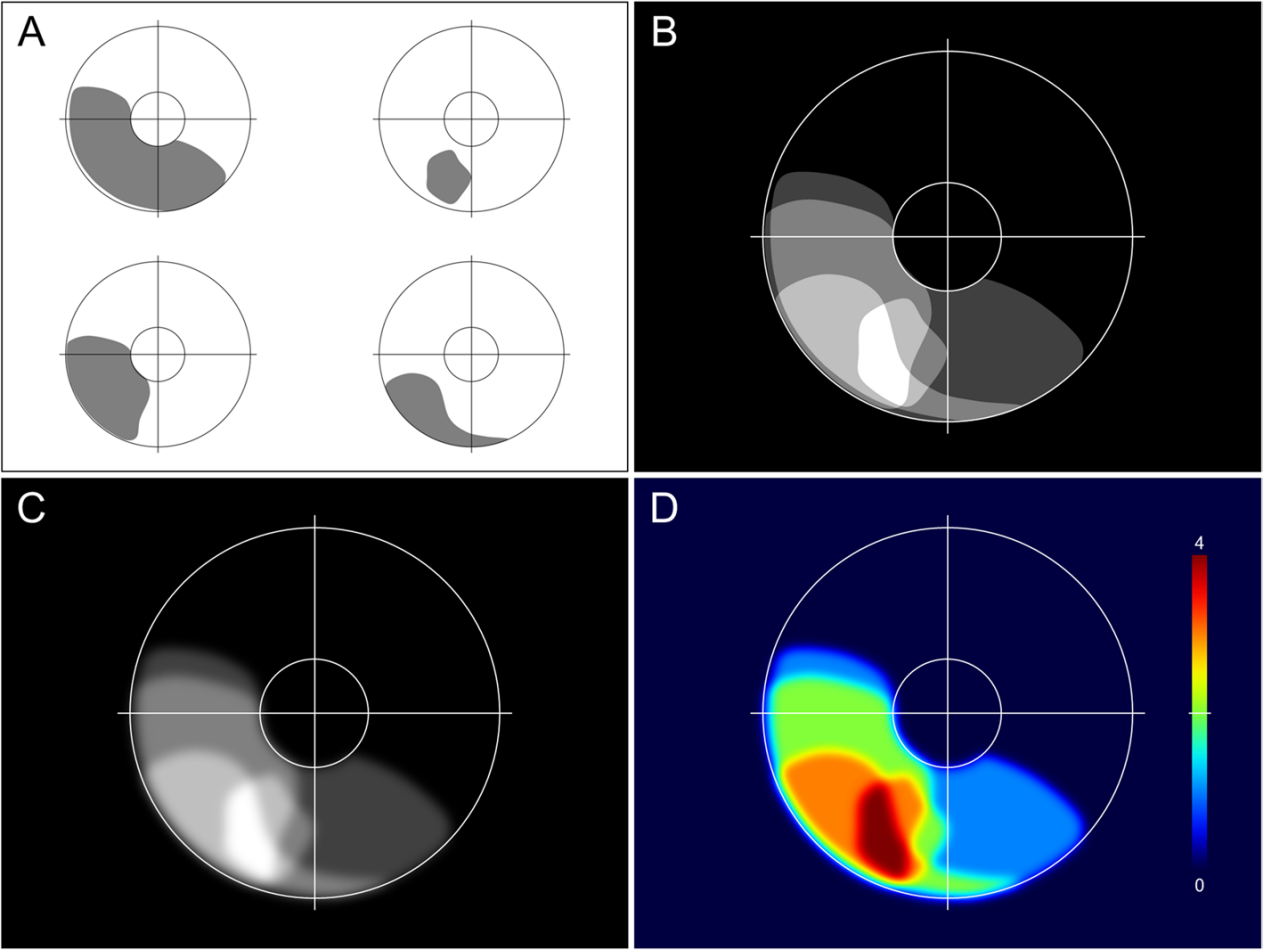
Illustration of the image processing to obtain the iris maps of tumour location and extent. **A** Four representative iris melanomas from the present study are drawn by the drawing tools of PowerPoint (Microsoft Corp., Redmond, WA, USA) on standardised iris drawing charts of right eyes. **B** A custom-made Matlab program (The MathWorks, Natick, MA, USA) is used to merge the original drawings and display the number of overlapping tumours by different shades of grey. **C** After individual filtering of the tumour drawings, the resulting image appears smoother with less disturbing sharp edges. **D** The image is then colour-coded with a blue-to-red gradient indicating increasing brightness. The top of the colour scale (dark red) represents the maximum number of overlapping tumours, which in this example is 4

Each iris melanoma was drawn on an iris drawing chart with a relative pupil diameter of 3.5 mm. The drawing tools of the computer software PowerPoint (Microsoft, Redmond, WA, USA) were used to convert all iris drawings into a database of identical two-dimensional charts. The iris charts of left eyes were flipped across their vertical axis, so that all eyes were displayed as right eyes. A custom-made Matlab program (The MathWorks, Natick, MA, USA), was used to merge, filter, and finally convert the collection of digital tumour drawings into an iris map, in which different colours indicated the number of overlapping iris melanomas (Fig. 1). Separate colour-coded maps were made for various subgroups of patients and tumours, where dark blue colour indicated areas without any tumours and dark red revealed the area with the highest number of overlapping iris melanomas.

### Statistical analysis

The Chi-square goodness-of-fit test was used to analyse the distribution of the iris melanomas under the null hypothesis that they are uniformly distributed in the iris and the assumption that each chart quadrant includes an equal area of the iris. The Fisher’s exact test was applied for comparison between two groups of patients or tumours with different characteristics (binary variables dichotomised at the median). Gender differences were assessed by the Fisher’s exact test for categorical variables and the Mann–Whitney test for continuous variables. The data were analysed using GraphPad software, (GraphPad Software Inc., San Diego, CA, USA). A two-sided *p*-value <0.05 was considered statistically significant.

## Results

### Patient and tumour characteristics

Twenty-nine patients (18 females and 11 males) met the inclusion criteria. The median age at the time of diagnosis for the entire cohort was 56 years (mean, 52 years; range, 18–80 years). The median age at diagnosis was 56 years (mean, 51 years) for females and 54 years (mean, 53 years) for males (*p* = 0.60). The right eye was involved in 17 patients and the left eye in 12 patients. The most common cause of referral was growth of a pre-existing naevus in 15 patients (52%), followed by a newly detected dark iris lesion in 9 (31%), pain secondary to elevated IOP in 3 (10%), and blurred vision due to spontaneous hyphaema in 2 (7%). The medical history indicated that the iris melanoma had arisen from a pre-existing naevus in 16 eyes (55%) and de novo in 13 eyes (45%). In females, the number of tumours developing from a naevus and de novo was 13 and 5, respectively, while the corresponding figures in males were 3 and 8 (*p* = 0.03). The median IOP was 14 mmHg (mean, 17.6 mmHg; range, 6–51 mmHg), and 5 patients (17%) presented with secondary glaucoma. The iris colour was blue in 25 patients (86%), green in 3 (10%), and unknown in 1 (3%).

**Fig. 2:**
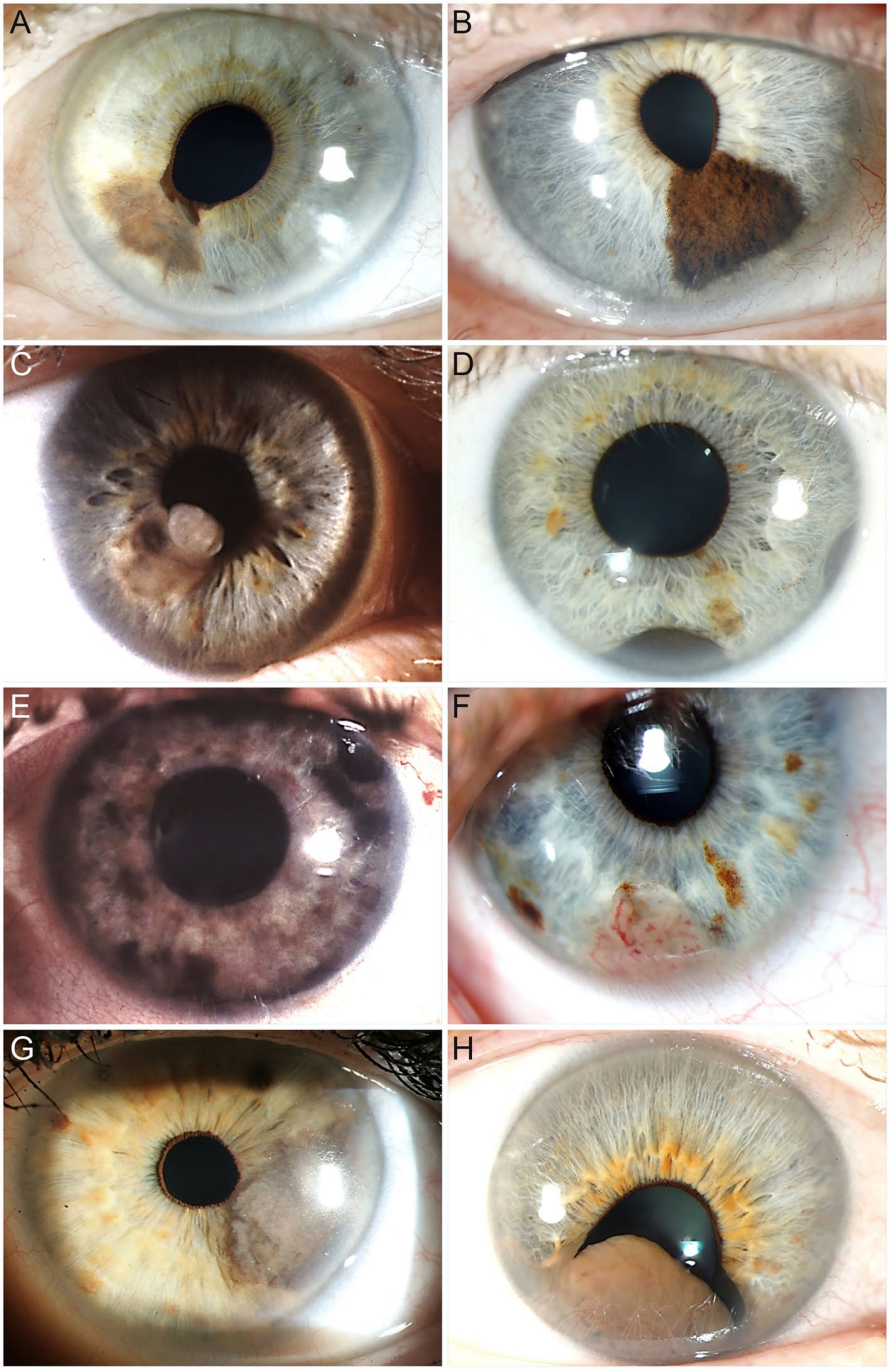
Slit-lamp photographs of selected study patients showing different morphological features of iris melanoma. **A** Flat, weakly pigmented iris melanoma with a small ectropion uveae. **B** Dome-shaped, markedly pigmented iris melanoma with an irregular surface. **C** Nodular, moderately pigmented iris melanoma. **D** Ring-shaped, markedly pigmented iris melanoma visible temporally and inferiorly. Gonioscopically, a multinodular pigmented mass was seen in the chamber angle from the 2.30 to 11 o’clock position. **E** Diffuse iris melanoma with multiple pigmented areas. **F** Amelanotic, vascularised iris melanoma. **G** Tapioca iris melanoma with multiple small, amelanotic nodules on the surface. **H** Large iris melanoma leading to pupillary distortion and ectropion uveae

The median largest tumour diameter was 5 mm (mean, 6.1 mm; range, 1.8–11.0 mm). In 7 tumours examined by UBM, the median tumour height was 2.0 mm (mean, 2.0 mm; range, 1.2–2.8 mm). The tumour was flat in 5 patients (17%), dome-shaped in 17 (59%), nodular in 5 (17%), ring-shaped in 1 (3%), and diffuse in 1 (3%). One tumour (3%) was amelanotic, while 4 (14%) were weakly, 5 (17%) moderately, 12 (41%) markedly, and 5 (17%) partially pigmented. Two tumours (7%) were classified as tapioca melanoma. Ectropion uveae and pupillary distortion were seen in 11 eyes (38%) and 15 eyes (52%), respectively. Examples of these tumour characteristics are shown in Fig. 2.

The diagnosis of iris melanoma was established by documented growth in 9 cases (31%) and by biopsy in 20 cases (69%), of which 13 (65%) were excisional biopsies, 4 (20%) were incisional biopsies, and 3 (15%) were performed with a vitreous cutter. Histopathology showed that 14 tumours (70%) were spindle cell type, 1 (5%) was epithelioid cell type, and 5 (25%) were mixed-cell type. Eleven patients (38%) underwent surgical iridectomy, and 12 patients (41%) were treated with brachytherapy (11 with a ruthenium plaque and 1 with an iodine-125 plaque). Three patients (10%) underwent primary enucleation, and 3 patients (10%) refused treatment. The median follow-up was 7 years (mean, 12 years; range, 1–28 years). Among the treated patients, there were no cases of local recurrence. One patient (3%) developed widespread metastatic disease including multiple liver lesions during follow-up. Eight patients (28%) were dead from other causes.

### Topographic tumour distribution

The melanoma involved the pupillary margin in 5 eyes (17%), the iris root in 10 eyes (34%), and both the pupil and peripheral iris in 10 eyes (34%). The anterior chamber angle was partly infiltrated by the melanoma in 13 (45%) eyes. Two patients (7%) presented with ciliary body involvement, of whom one also had extrascleral tumour extension.

**Fig. 3:**
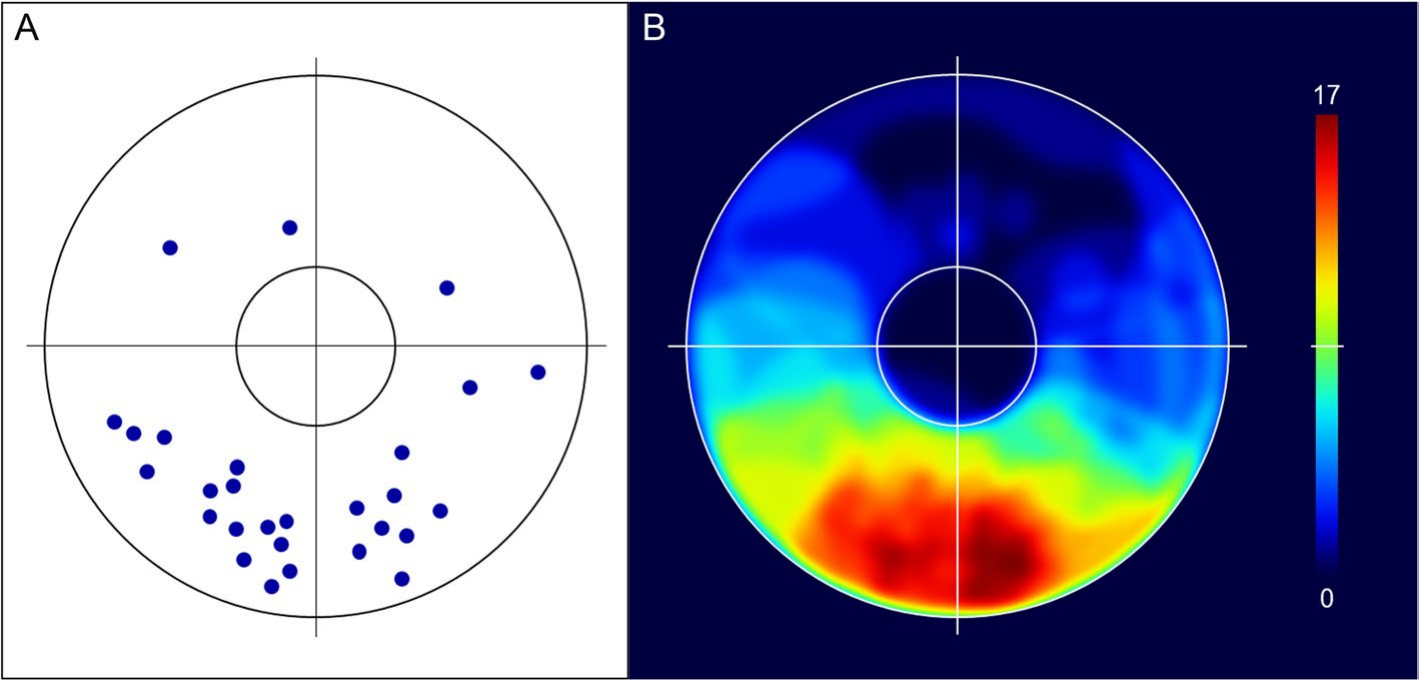
Iris charts displayed as right eyes, so that the left part of the figures represents the temporal side of the iris. The horizontal and vertical lines passing through the pupil centre divide the iris into quadrants and hemispheres. **A** Geometric centres of 28 iris melanomas indicated by blue dots. **B** Merged iris drawings showing the pattern of tumour extent of 29 iris melanomas. The colours on the chart indicate the number of overlapping iris melanomas according to the scale bar to the right. The top of the scale (dark red) represents the maximum number of overlapping tumours (17), and the bottom (dark blue) indicates no tumours

The topographic distribution of the geometric centres of 28 iris melanomas is illustrated in Fig. 3A. One patient with a diffuse iris melanoma were excluded from this distribution analysis. There was a significant superoinferior asymmetry in the distribution of the tumour centroids, as 3 (11%) were located in the superior and 25 (89%) in the inferior iris hemisphere (*p* < 0.0001). The distribution between the temporal and nasal iris hemisphere was 17 (61%) and 11 (39%), respectively (*p* = 0.26).

In females, the number of tumour centroids in the temporal and nasal hemisphere was 14 and 4, respectively, while the corresponding figures in males were 3 and 7 (*p*  =  0.02). The hemispherical distribution of the tumour centroids did not differ significantly between patients in the age groups <56 years and ≥ 56 years, tumours that had arisen from a pre-existing naevus and de novo, tumours with a largest diameter of <5 mm and ≥ 5 mm, or between tumours grouped according to the other investigated tumour characteristics (Table 1).

**Table 1:** Topographical distribution of the tumour centroids of 28 iris melanomas in various fundus hemispheres, according to binary patient and tumour characteristics

Binary variables	Eyes	Hemisphere (***n***)					
	***n***	Temporal	Nasal	p*	Upper	Lower	p*
Female	18	14	4	**0.02**	2	16	1.00
Male	10	3	7		1	9	
Age < 56 years	13	9	4	0.46	1	12	1.00
Age ≥ 56 years	15	8	7		2	13	
Naevus-associated melanoma	16	10	6	1.00	2	14	1.00
De novo melanoma	12	7	5		1	11	
Largest tumour diameter < 5 mm	13	6	7	0.25	3	10	0.09
Largest tumour diameter ≥ 5 mm	15	11	4		0	15	
Ectropion uveae	11	6	5	0.70	1	10	1.00
No ectropion uveae	17	11	6		2	15	
Secondary glaucoma	4	3	1	1.00	0	4	1.00
No secondary glaucoma	24	14	10		3	21	

* *p*-values calculated using the Fisher’s exact test

**Fig. 4:**
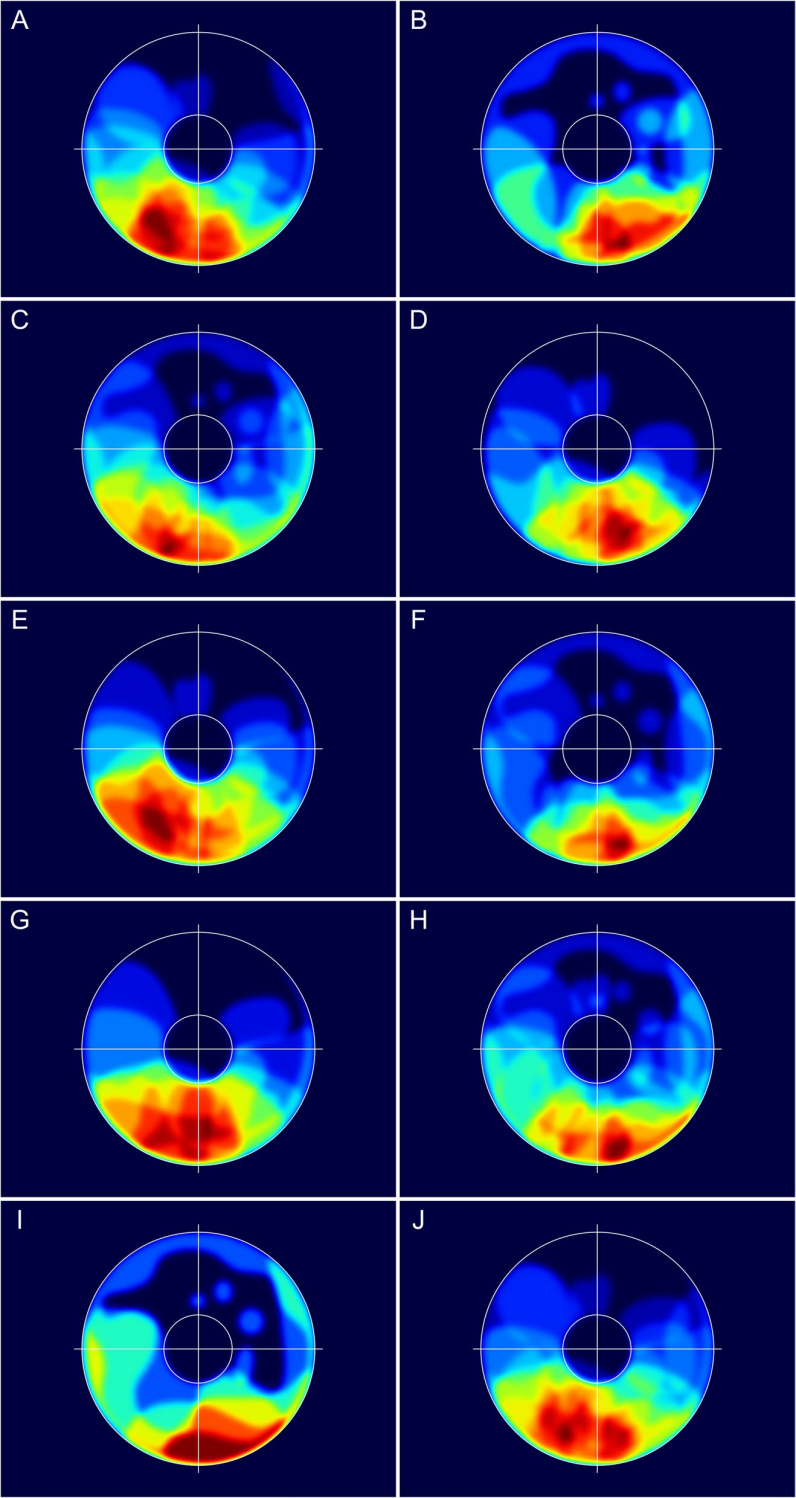
Merged iris drawings showing the patterns of tumour extent according to various patient and tumour characteristics. The image details and colour scaling are as described in Fig. 3. Note that the maximum number of overlapping tumours differs between the images. The following numbers in parentheses refer to the maximum number of overlapping tumours. **A** Females (12); **B** males (7); **C** patients <56 years of age (11); **D** patients ≥56 years of age (10); **E** tumours arising from a pre-existing naevus (10); **F** tumours arising de novo (10); **G** Tumours with ectropion uveae (9); **H** tumours without ectropion uveae (10); **I** eyes with secondary glaucoma (5); **J** eyes without secondary glaucoma (13)

The patterns of tumour extent, visualised by the computationally merged iris charts, corresponded well with the regional distribution of the tumour centroids. The area with the highest number of overlapping tumours was located inferiorly and slightly temporal in the iris, and the upper nasal quadrant was the area with the fewest overlapping tumours (Fig. 3B). The iris melanomas were located more temporally in females compared with males (Fig. 4A,B). Similarly, there was a clear shift towards a more temporal location of the iris melanomas in younger patients compared with the older ones (Fig. 4C,D), and in tumours arising from a pre-existing naevus compared with those arising de novo (Fig. 4E,F).

The merged iris charts also demonstrated that melanomas leading to ectropion uveae were located in closer proximity to the pupillary margin than in eyes without ectropion uveae (Fig. 4G,H). In eyes with secondary glaucoma, the tumours presented with a ring-like growth pattern in the peripheral iris and angle region (Fig. 4I,J).

## Discussion

This study provides detailed clinical and topographical data on patients with newly diagnosed iris melanoma over a 30-year period. It is generally believed that there is no gender predilection for the development of iris melanoma [7]. In the present study, there was a slight predominance of female patients (62%), and a similar female predominance has also been found by others [10, 11]. The mean age at the time of diagnosis was 52 years, which is in accordance with many other studies showing that patients with iris melanoma are about a decade younger at presentation than those with posterior uveal melanoma [3, 9, 12]. In an earlier study conducted in the same population, the mean age at diagnosis of patients with posterior uveal melanoma was 64 years [13]. This difference in age distribution may be due in part to the fact that iris melanomas are more easily detected by clinical examination [5]. The generally more benign nature of iris melanoma compared with posterior uveal melanoma was confirmed histologically by their predominant spindle cell component and by the low rate of metastatic spread during the long follow-up period.

Previous studies have consistently shown that the majority of iris melanomas are located in the lower half of the iris [9, 7, 11, 12, 14]. This differs significantly from the meridional locations of both ciliary body and choroidal melanomas, which have been shown to be equally distributed between the superior and inferior hemispheres of the eye [7, 15, 16]. In the present study, 89% of the tumour centroids were located in the inferior iris hemisphere, and both the numerical distribution and the merged iris drawings demonstrated that iris melanomas have a predilection for the inferotemporal iris quadrant. Previous studies on the nasotemporal distribution of iris melanoma are inconsistent, with some reporting an increased frequency in the temporal part of the iris [7, 17–19], and others reporting a uniform horizontal distribution [3, 20, 21]. The characteristic localisation of iris melanomas has been linked to exposure to ultraviolet (UV) radiation [7, 22–24]. As the superior orbital rim and upper eyelid shield the superior part of the iris and the nose shadows the nasal part, the inferotemporal quadrant is probably the most sun exposed iris area and the superonasal quadrant the least [25]. Iris naevi also have a predilection for the inferior iris [9, 18, 26], and this location has been identified as a predictive risk factor for malignant transformation into melanoma [14]. In a recent study, Schwab et al. found that most iris freckles are located in the inferotemporal quadrant [25]. Although iris freckles, unlike iris naevi, are considered to lack malignant potential [7, 9], the presence of iris freckles may be indicative of sunlight exposure and serve as a potential biomarker for chronic sun damage [25]. The role of sunlight exposure in the pathogenesis of iris melanoma is further supported by the observations of an increased prevalence of UV light-induced mutations in these tumours [24, 27–29].

An unexpected finding in our study was the significant gender difference in tumour localisation, where more iris melanomas were located temporally in females compared with males. A similar and more temporal pattern of tumour extent was also seen in the younger age group and among iris melanomas originating from a previously known naevus, and it is tempting to speculate that there could be some gender variation in behaviour or unknown biological relation between these features. There was no statistically significant age difference between genders, but females had a significantly higher proportion of melanomas arising from a pre-existing naevus than males. In a large study comparing gender differences in uveal melanomas, Damato & Coupland found that uveal melanomas in males tended to be larger and located more posteriorly than in females, and that females with iris melanoma presented at a younger age than males [10]. In the present study, no clear topographical differences were found between the other investigated tumour characteristics, except for ectropion uveae and secondary glaucoma. As to be expected by the secondary effects of tumour growth, iris melanomas associated with ectropion uveae were in closer proximity to the pupillary margin and tumours leading to secondary glaucoma showed a more peripheral and circumferential growth pattern.

The main limitations of the present study are its retrospective design and relatively small sample size, while its strengths include the strict inclusion criteria, the high proportion of biopsy proven tumours, and the long follow-up period. The distribution analysis implies that iris melanomas exhibit an isotropic growth mode directed symmetrically from the geometric tumour centre, which may not always be the case. This is compensated for by the merged iris drawings, which merely illustrate the frequency with which certain iris regions are affected by the tumours independent of their growth pattern.

## Conclusions

Both the distribution of the tumour centroids and the merged iris charts demonstrate that the inferotemporal iris quadrant is the most frequent location of iris melanoma. Iris melanoma tends to be more temporally located in females, who compared with males are also more likely to have melanoma arising from a pre-existing naevus. The study shows that the pattern of tumour extent differs among various clinical and morphological subgroups of iris melanoma, which calls for further studies to better understand the mechanisms behind growth and malignant transformation of iris melanocytic lesions.

## Data Availability

The datasets used during the current study are available from the corresponding author on reasonable request.

## Abbreviations

IOP: Intraocular pressure
UBM: Ultrasound biomicroscopy
UV: Ultraviolet
